# Functional Magnetic Resonance Imaging of Cognitive Control following Traumatic Brain Injury

**DOI:** 10.3389/fneur.2017.00352

**Published:** 2017-08-04

**Authors:** Randall S. Scheibel

**Affiliations:** ^1^Michael E. DeBakey Veterans Affairs Medical Center, Houston, TX, United States; ^2^Department of Physical Medicine and Rehabilitation, Baylor College of Medicine, Houston, TX, United States

**Keywords:** traumatic brain injury, cognitive control, executive function, functional magnetic resonance imaging, default mode network

## Abstract

Novel and non-routine tasks often require information processing and behavior to adapt from moment to moment depending on task requirements and current performance. This ability to adapt is an executive function that is referred to as cognitive control. Patients with moderate-to-severe traumatic brain injury (TBI) have been reported to exhibit impairments in cognitive control and functional magnetic resonance imaging (fMRI) has provided evidence for TBI-related alterations in brain activation using various fMRI cognitive control paradigms. There is some support for greater and more extensive cognitive control-related brain activation in patients with moderate-to-severe TBI, relative to comparison subjects without TBI. In addition, some studies have reported a correlation between these activation increases and measures of injury severity. Explanations that have been proposed for increased activation within structures that are thought to be directly involved in cognitive control, as well as the extension of this over-activation into other brain structures, have included compensatory mechanisms, increased demand upon normal processes required to maintain adequate performance, less efficient utilization of neural resources, and greater vulnerability to cognitive fatigue. Recent findings are also consistent with the possibility that activation increases within some structures, such as the posterior cingulate gyrus, may reflect a failure to deactivate components of the default mode network (DMN) and that some cognitive control impairment may result from ineffective coordination between the DMN and components of the salience network. Functional neuroimaging studies examining cognitive control-related activation following mild TBI (mTBI) have yielded more variable results, with reports of increases, decreases, and no significant change. These discrepancies may reflect differences among the various mTBI samples under study, recovery of function in some patients, different task characteristics, and the presence of comorbid conditions such as depression and posttraumatic stress disorder that also alter brain activation. There may be mTBI populations with activation changes that overlap with those found following more severe injuries, including symptomatic mTBI patients and those with acute injuries, but future research to address such dysfunction will require well-defined samples with adequate controls for injury characteristics, comorbid disorders, and severity of post-concussive symptoms.

Traumatic brain injury (TBI) is a neurological insult of major public health significance with over 1.7 million new injuries each year among Americans under the age of 35 (1). Numerous studies, most of which have been conducted with moderate-to-severe TBI due to blunt head trauma, have reported findings consistent with a mixed and highly heterogeneous neuropathology that may include multifocal or diffuse axonal injury, as well contusions and other focal lesions (2). Additional injury may occur as a result of edema, herniation, hemorrhage, ischemia, inflammation, and excitotoxic processes (2, 3). Structures and connections of the frontal and limbic regions have been said to be especially vulnerable to these various pathological processes (3, 4). Executive functions are highly dependent on the integrity of this neural substrate, and it is not surprising that such functions, including cognitive control, are often impaired following TBI (5–7).

Cognitive control allows for flexibility in human thought and behavior and may be defined as the ability to pursue task-related goals in the presence of conditions that include conflicting information or interference, prepotent response alternatives, or the need to interrupt or switch an ongoing activity (8–10). A common factor in all of these situations is the top-down direction, or biasing, of cognition and this is necessary for information processing and behavior to adapt from moment to moment depending on task requirements and performance (8, 11, 12). Cognitive control relies upon the active maintenance of neural activity associated with the internal representation of goals and task-related rules or contingencies (11–13). However, it is a complex construct that likely includes multiple component processes, some of these processes overlap with those of other executive functions (e.g., working memory), and it contributes to performance on various high level cognitive tasks, including those representing domains such as attention, memory, and language (8–10, 14).

Although prefrontally guided top-down direction is critical for cognitive control and other executive functions, the prefrontal cortex (PFC) is only one of several structures that contribute to cognitive control (15). Another important structure is the anterior cingulate cortex, which is thought to monitor performance and internal bodily states associated with task-related reward conditions, to determine whether task performance is adequate, and to signal to the dorsolateral PFC when mental effort or top-down direction needs to be increased (11, 15–17). Some anterior cingulate functions, including the detection of states associated with reward and expected outcomes, likely depend on distant connections with structures such as the insula (17). These various structures may be vulnerable to disconnection associated with diffuse axonal injury and other TBI-related neuropathology (18–20).

Functional magnetic resonance imaging (fMRI) provides an indirect measure of neural activity and has the potential to reveal changes in brain function associated with neuropathology, including alterations following TBI (21, 22). One powerful application of this method is the use of fMRI paradigms to examine brain activation during cognitive tasks (22), including those which place a demand upon executive functions such as cognitive control. This type of research has the potential to reveal relationships between specific cognitive impairments and dysfunction within the underlying neural substrate, to provide a neuroimaging marker that may contribute to differential diagnosis, and to lead to the development of methods to track changes in brain activity associated with recovery and treatment (23). Cognitive control is a high level function that is critical for the completion of many complex and non-routine tasks (8, 11). Despite the importance of this topic and the incredible potential offered by fMRI research, only a few studies have examined changes in cognitive control-related activation following TBI, and these have often suffered from various methodological limitations. The purpose of this article is to provide an overview of that existing research, to discuss findings that contribute to our understanding of how cognitive control may be impaired following TBI, and to provide some suggestions to improve future research and increase its relevance.

Although fMRI research has also investigated working memory and other executive functions following TBI (24, 25), the current review will focus on cognitive control by examining fMRI studies that have specifically addressed the top-down direction of cognition and related cognitive control processes (e.g., performance monitoring). This research has employed fMRI paradigms adapted from common clinical measures of cognitive control, such as the Stoop Test (26), as well as experimental procedures developed specifically for the purpose of acquiring fMRI data [e.g., Ref. (27)]. Studies using paradigms that assess other functions, such as working memory or attention, are also included within this review if they had incorporated procedures to investigate top-down control [e.g., Ref. (28)]. Some had utilized a block design approach [e.g., Ref. (29)], whereas others had employed event-related fMRI [e.g., Ref. (30)]. A major feature of block design fMRI paradigms is that this method combines images acquired across an entire block of trials, which then prevents the separation of images acquired within a block to examine activation relative to different types of stimuli or responses (31). Event-related designs have the advantage of allowing the examination of images at the trial level, including the ability to isolate correct or incorrect responses, but these designs typically have less statistical power (31). It is also possible to capitalize upon some of the advantages of both approaches by employing a mixed design (32).

## Initial Studies of Cognitive Control Activation Following Moderate-To-Severe TBI

One of the first published reports of cognitive control-related activation following TBI involved a single-case study and used a block design stimulus–response compatibility paradigm (27). This patient had sustained a severe TBI 1 year earlier and, although there was no evidence of focal injury on CT scans performed on the day of his motor vehicle accident, at the time of the study he had ventricular dilation consistent with severe diffuse injury and was moderately disabled. fMRI was performed with the Arrows Task, which is a simple fMRI cognitive control paradigm that requires the use of a hand held button device to respond to blue and red colored arrows that are presented on a screen, one at a time, with each arrow pointing to the left or to the right. During stimulus compatible blocks, the arrows are blue and the correct response is to press the button that is on the same side the arrow is pointing toward, while stimulus incompatible blocks include only red arrows and then the correct response is to press the button on the opposite side. The contrast of interest involved the subtraction of blood oxygen level dependent (BOLD) signal during stimulus compatible (i.e., blue arrow) blocks from that acquired during the stimulus incompatible (i.e., red arrows) blocks. Whole brain image analyses and the examination of activation within medial and lateral frontal regions of interest (ROIs) indicated greater and more distributed activation for this patient, relative to a healthy comparison subject. These results were generally consistent with those of an early investigation of computational working memory which also found regionally dispersed activation following moderate-to-severe TBI (24), but they differed from those of a couple of subsequent studies which specifically examined cognitive control within small TBI samples (33, 34).

Easdon et al. (33) imaged a group of five patients with moderate-to-severe TBI who had been injured in motor vehicle accidents 14–21 months earlier. Their event-related paradigm measured response inhibition using a “go-stop” task that required the response to be withheld whenever the color of target letters changed from white to red. In addition to the letters, the task included a number of null event trials without a target letter (i.e., fixation point only) and this design allowed the examination of several separate contrasts, including stop-signal versus null events. The TBI subjects were matched to a group of five healthy comparison subjects on both go-signal reaction time and the number of inhibitions, but the injured group had more omission errors. Between-group fixed-effects analyses indicated greater activation for control subjects during stop trials where the response was successfully withheld, relative to the TBI group, within dorsolateral PFC. Similarly, during correct go-signal responding the healthy subjects had greater activation than the TBI patients within bilateral PFC, left visual cortex, and the anterior cingulate gyrus. Another early investigation (34) also reported reduced cognitive control-related activation within anterior cingulate gyrus in five patients who had sustained a severe TBI 1–7 years earlier. This study included a comparison group of 11 healthy subjects and had used an fMRI block design adaptation of the Stroop Test with a contrast comparing activity during the cognitive task with that of an awake, eyes open resting condition. A between-group analysis showed that the TBI and healthy subjects did not differ on task performance, but no direct between-group comparisons were reported for the fMRI activation data. Instead, separate within-group fixed-effects analyses were performed, and inspection of the activation patterns was said to indicate decreased anterior cingulate activation within the TBI group.

These early investigations all found that TBI was associated with altered cognitive control-related activation, but none had used an adequately sized sample or performed a random effects between-group analysis. Moreover, the fMRI data presented by Soeda et al. (34) did not include any formal between-group comparisons and, consequently, their findings may reflect higher variability in the TBI activation data or reduced power for the TBI within-group analysis due to its substantially smaller sample size relative to the comparison group. Thus, there was a need for additional research with between-group comparisons and random-effects analyses, as well as the ability to address other factors such as task performance, the influence of demographic variables and other subject characteristics, and differences in injury severity.

## Global Activation Pattern Changes Following Moderate-To-Severe TBI

A pair of studies using fMRI cognitive control data with random-effects whole brain image analyses compared patients with moderate-to-severe TBI and those with only orthopedic injury (35, 36). Previous fMRI research had imaged healthy uninjured subjects for comparison with others who had sustained a TBI, but this approach fails to control for host factors that predispose to injury (e.g., risk taking) or stress associated with trauma and medical treatment. In the first of these studies, 14 patients with moderate-to-severe TBI were compared to 10 with orthopedic injury (35) using the same block design fMRI paradigm as the original Arrows Task case study (27). These groups did not differ in age, education, or estimated pre-injury IQ and prescan training was employed to elevate the TBI patients’ performance so that it approximated that of the comparison group. During scanning, the task accuracy was marginally higher in the orthopedic injury patients, but reaction time did not differ between the groups. In a random-effects between-group analysis, the TBI patients had greater activation bilaterally within medial frontal structures and the cingulate gyrus. For the orthopedic injury group, there was a significant positive correlation between accuracy and activation within the parietal and medial frontal lobes, but this correlation was not significant when examined within the TBI group. Instead, acute severity of the TBI, as assessed by the first available post-resuscitation Glasgow Coma Scale (GCS) score, had a negative correlation with activation within midline cortical structures (e.g., anterior cingulate gyrus) and deep subcortical structures such as the thalamus and basal ganglia. These findings provided preliminary evidence for greater activation in association with lower GCS scores and raised the possibility that the between-group results may have been driven by those subjects with the most severe brain injuries. In addition, differences in the regression results for performance accuracy and activation were interpreted as indicating a breakdown in the normal relationship between accuracy and brain function following TBI, such that increasing levels of activation were not associated with improved performance, but this study had used a relatively small sample for the types of analyses that were completed and the regression findings were considered to be tentative.

A second study was performed using Arrows Task data from some of the same subjects to provide a more in-depth examination of the relationship between cognitive control-related activation and acute injury severity (36). The number of TBI subjects was increased to 30, and the GCS total score was used to divide these into subgroups with moderate (GCS 9–15), severe (GCS 5–8), or very severe (GCS 3–4) injury. Although moderate severity is often considered to be associated with a GCS score under 13, assignment to this group was permitted for those with better scores if they had a focal injury on computerized tomography performed at the time of injury [i.e., complicated mild TBI (mTBI)] (37). Comparisons among these subgroups indicated no significant differences between the orthopedic injury and moderate TBI patients. However, in comparisons with severe injuries, there was greater activation in clusters centered within posterior midline areas, such as the medial parietal and occipital lobes, and in those patients with the most severe injuries this elevated activation (i.e., over-activation) also extended into lateral cortex. Additional structures that were engaged only in patients with very severe TBI included the left inferior and superior parietal lobules and a posterior region of left lateral frontal cortex, areas which are thought to be involved in language functions and various high level cognitive skills. These results suggest the possibility that more extensive cortex may be recruited to overcome TBI-related cognitive impairment and, in the case of the most severe injuries, there may be greater reliance upon language or verbal strategies while performing the task.

The finding that cognitive control-related activation is greater following TBI is generally consistent with the results of other neuroimaging studies that have examined the influence of neurological disorders on cognitive activity, including the engagement of working memory. Hillary (25) observed that the vast majority of these studies have reported greater recruitment of neural resources in clinical samples, including those with TBI, but also mentioned that the cause of this increased activation has been variously attributed to mechanisms such as neural inefficiency, compensation, and brain reorganization. The consideration of task performance is critical for the interpretation of fMRI findings and for relating activation changes to underlying cognitive or neural mechanisms, especially when a healthy group is compared to a clinical group with impairment (22). When performance is unbalanced, it is then unclear whether differences in task-related activation occur because of difficulty complying with the instructions, failure to implement an effective cognitive approach or strategy, or an inefficiency or other change in the utilization or organization of the underlying neural substrate (22, 38). To address this issue, many fMRI studies have equated task accuracy between their groups, often using procedures such as subject matching or titrated prescan training, or they have isolated correct response trials or included accuracy as a variable within the statistical design (22, 39). However, Hillary (25) noted that reaction time is also an important aspect of performance and that neuroimaging studies have often under-examined this variable. He proposed that, when pressured to maintain a high degree of accuracy, both impaired and intact subjects may adjust their response speed and that this is actually a normal mechanism that is supported by the PFC. Increased prefrontal activation in patients with neurological disorders may thus represent the same basic cognitive control process that is typically engaged in healthy subjects to address increased task demands, but because of their pathology the patients may recruit such neural resources at a lower threshold. According to this particular view, the elevated prefrontal activation found in clinical samples should be considered to be a compensatory neural mechanism only if it can be shown to bolster task performance, otherwise it is likely to reflect only the engagement of a normal cognitive process and its prefrontal substrate.

Elevated cognitive control-related activation has also been observed in structures that extend well beyond the PFC, including subcortical and posterior cortical brain structures (36, 40), and these activation changes following TBI may be associated with other mechanisms that impact the functioning of widely distributed neural networks. One possible mechanism includes limitations or inefficiencies which the patient may be able to overcome for some period of time, but only through the exertion of increased effort that requires the allocation of greater resources and ultimately results in cognitive fatigue (41). Kohl et al. (29) examined whether cognitive fatigue contributes to TBI-related activation increases during tasks demanding cognitive control. These investigators employed a block design fMRI paradigm based on the Symbol Digit Modalities Test (SDMT) (42) and their contrasts compared identical SDMT blocks from different time points during the same scan session. Although the healthy subjects had better reaction time during the SDMT, for both groups the response speed increased as they performed the task and accuracy levels always remained high. As the task progressed the healthy subjects exhibited activation decreases within several structures, including the anterior cingulate and middle frontal gyri, superior parietal cortex, and the basal ganglia. However, despite having high performance and similar improvements in response speed, as the TBI subjects performed the task they exhibited activation increases. Decreased activation within the healthy group may have resulted from more efficient utilization of neural resources and less effortful cognitive processing as they became familiar with the task (12, 43), but patients with TBI instead exhibited increased activation suggestive of increased effort and cognitive fatigue. Although these fMRI findings appear to support the need for greater cognitive effort to maintain performance following brain injury, whether or not the subjects actually experienced subjective feelings of increased effort or fatigue was not explored.

**Figure 1:**
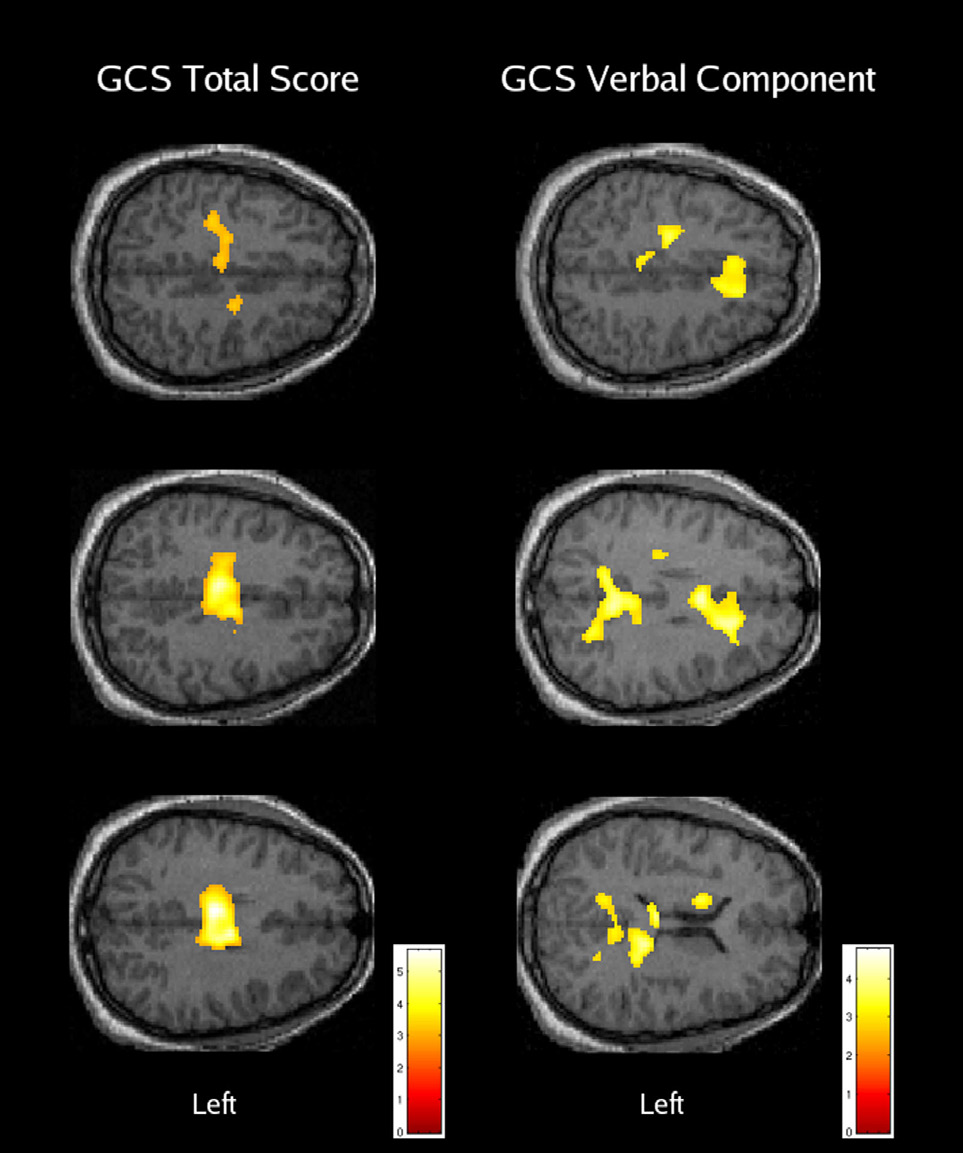
The relationship between cognitive control-related activation and acute traumatic brain injury severity. Areas with a significant negative regression coefficient between brain activation and the Glasgow Coma Scale (GCS) total score (left column) or verbal component score (right column) are overlaid on axial anatomical images from a typical orthopedic injury patient [reused with permission from Ref. (36)].

In summary, the research addressing global changes in cognitive control-related activation following moderate-to-severe TBI has been limited, but there is support for the presence of increased and more dispersed activation in patients with severe TBI (35, 36). Although a number of studies included patients with moderate TBI (33, 35, 36), the only comparison that specifically addressed the moderate severity level did not reveal significant differences relative to a well-matched group of subjects with orthopedic injury alone (36). Samples for the individual subgroup analyses performed by Scheibel et al. (36) were relatively small, but there were no instances where the orthopedic patients or a less severely injured TBI subgroup had greater activation than a subgroup with more severe TBI. In addition, image regression analyses (*n* = 30) conducted through the range of moderate to “very severe” injury indicated significant negative correlations between activation and both GCS total and GCS verbal component scores (see Figure 1). Overall, these findings provide support for elevated activation that increases in association with greater diffuse brain injury, but only when the initial GCS total score is 8 or less (i.e., severe TBI). Research also provides some evidence for several neural mechanisms that may potentially underlie over-activation following TBI, including alterations in the response pattern (25), neural inefficiency (36, 44), increased effort or cognitive fatigue (29), and the recruitment of additional brain structures associated with compensatory cognitive functions (36).

## Parcellation of Cognitive Control and Moderate-To-Severe TBI

The ability to relate activation changes to specific cognitive and neural mechanisms requires more complicated experimental designs and integration with other data reflecting neuropathology, such as measures from diffusion tensor imaging (DTI) (18, 31). Although studies using block design fMRI and whole brain image analyses provide evidence for elevated activation, they offer no additional information about differences associated with correct or incorrect performance, behavioral adjustments and activation changes in response to error detection, or the relationship between activation and specific types of task stimuli. The use of event-related fMRI paradigms offers such an opportunity (31). Sozda et al. (30) employed an event-related, task-switching cued-Stroop paradigm to study cognitive control in a sample of 10 patients with severe TBI and 12 healthy comparison subjects. They used a contrast comparing BOLD signal during incorrect and correct response trials to examine error-related activation. Relative to their healthy subjects, the TBI group had significantly more errors during the incongruent task condition, more extensive error-related activation, and a higher level of such activation within the anterior cingulate cortex. The activation changes did not appear to be associated with improved task performance since the TBI patients had committed more errors. These findings were considered to be consistent with the disruption of a specific component of cognitive control following severe TBI, error-related processing, along with associated dysfunction within the anterior cingulate gyrus. Performance monitoring is thought to be an important function of the anterior cingulate cortex that serves not only to detect errors but to also signal the need for top-down behavioral adjustments (e.g., slower responding) (45). Impaired error-related processing following TBI may interfere with the ability to implement these strategic adjustments to improve performance.

Another approach for examining performance monitoring was employed by Ham et al. (46), who as part of a larger study (18, 20) used a cognitive control task outside of the scanner to identify TBI patients who had difficulty recognizing and correcting their own errors. These investigators assessed a sample of mild to severe TBI patients using a behavioral measure of performance monitoring, the stop-change task (47). The resulting data were then used to assign subjects to a low performance monitoring group consisting of 18 TBI patients who had difficulty with error correction on the stop-change task, relative to healthy subjects, and a high performance monitoring group with 30 TBI patients. All patients with low performance monitoring had sustained a moderate-to-severe TBI, as a group they underestimated their level of disability on the Frontal Systems Behavior questionnaire (48), and they were described as having broad deficits in attention. However, they were similar to the high performance monitoring TBI group on measures of memory, verbal and non-verbal reasoning, anxiety, and depression.

Ham et al. (46) scanned both of these TBI groups and 25 healthy subjects using an event-related stop-signal fMRI paradigm and examined a contrast comparing incorrect stop trials with go trials, an approach which provided a measure of neural activity during error-related processing. When compared to healthy subjects, the low performance monitoring TBI group had increased error-related activation within the bilateral insula and the parietal operculum and, relative to TBI patients with high performance monitoring, they exhibited increases within the left insula and parietal operculum. In contrast, the high performance monitoring TBI group had greater error-related activation than the healthy control group within the right middle frontal gyrus, left caudate nucleus, and bilateral putamen. Ham et al. (46) speculated that increased error-related activation in patients with high performance monitoring may reflect a type of functional compensation. Overall, their results were said to be consistent with impaired performance monitoring in some patients with moderate-to-severe injury that is part of a more general deficit in self-awareness, dysfunction involving the insula, and an inability to effectively engage compensatory mechanisms. These investigators also examined a contrast comparing correct stop trials with correct go trials and found no significant activation differences between the two TBI performance monitoring groups, but activation differences were found when all TBI patients were compared to the healthy control group (18). Those findings, as well as the relationship between fMRI activation and other neuroimaging measures of TBI pathology (18, 20), are discussed in the next section.

An examination of TBI-related dysfunction and multiple processes associated with cognitive control was also performed by Olsen et al. (32), who used a mixed design Not-X continuous performance task (CPT) fMRI paradigm with 62 patients who had sustained a moderate-to-severe TBI and 68 healthy comparison subjects. An event-related element of their design employed a contrast comparing non-target and target trials, while the block design portion compared separate task and rest (i.e., fixation only) blocks. This design separated the investigation of adaptive cognitive control processes, assessed using event-related fMRI, from more stable processes examined using block design procedures. Sustained attention and task-set maintenance are examples of stable top-down processes that are thought to be dependent on dorsolateral PFC and are effortful, operate on a long time scale, and are prone to decline with greater time on task (TOT) (49). Such processes are likely to be especially vulnerable to cognitive fatigue, which is a mechanism that previous research had implicated in TBI-related cognitive impairment (29). Adaptive processes, in contrast, have been said to be more dependent on the anterior cingulate cortex and operate on a shorter timeframe, are more automatic and reactive, and are less sensitive to the effects of fatigue (50).

**Figure 2:**
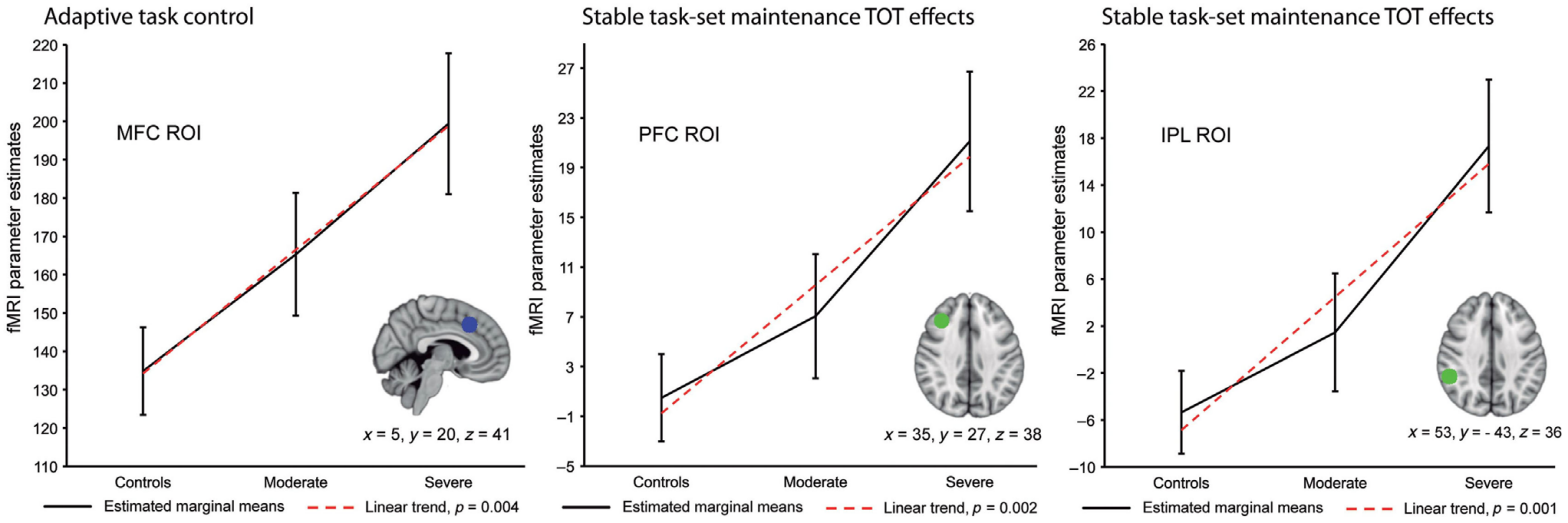
Region of interest (ROI) analyses for adaptive and stable cognitive control processes across healthy subjects and patients with moderate or severe traumatic brain injury (TBI). This figure shows the results of planned polynomial contrasts following statistically significant Multivariate Analyses of Covariance. Results are adjusted for age, education, and task performance. There was a significant linear trend within the medial frontal cortex (MFC) ROI for adaptive cognitive control processes. For stable cognitive control processes a significant linear trend was found for time on task (TOT) effects within the right lateral prefrontal cortex (PFC) and inferior parietal lobule (IPL) ROIs. Coordinates are from the Montreal Neurological Institute 152 T1 template [reused from Ref. (32) by permission of Oxford University Press].

During the Not-X CPT, there were no significant between-group differences for task performance and, for whole brain image analyses, activation did not differ for either the event-related or block design contrasts. However, an *a priori* analysis indicated greater activation for the TBI patients within an ROI in the medial frontal cortex (MFC), which also included the anterior cingulate gyrus, during adaptive but not stable cognitive control processes. These activation increases were found to be greater in those TBI patients with severe TBI (see Figure 2). In addition, activation changes associated with TOT were assessed by comparing activation during the early portion of the scan session relative to the last. For adaptive control processes (i.e., results from event-related fMRI), there were no between-group differences for TOT, but for stable cognitive control processes the TBI patients had increased activation within right lateral prefrontal and parietal areas with longer task duration. Within the right PFC, these TOT effects were noted to be greatest in TBI patients with severe injury. Also, for the TBI patients, partial correlation coefficients were calculated between activation and scores on the adult version of the Behavioral Rating Inventory of Executive Function (BRIEF) (51), which is a self-report measure of cognitive control problems, while statistically controlling for GCS total score, task performance, and both age and education. There was a negative relationship between BRIEF scores and stable process activation within medial frontal, right inferior parietal, and right PFC indicating that higher activation is associated with better cognitive control.

Studies that have examined separate component processes of cognitive control reveal several different changes following TBI, including impaired error-related processing (30, 46), medial frontal dysfunction during adaptive cognitive control processes, and higher frontal and parietal activation in association with effortful attention and task maintenance (i.e., stable cognitive control processes) as a function of greater TOT (32). There is also some evidence for a relationship between better cognitive control in day-to-day situations (i.e., BRIEF-Adult scores), as well as elevated stable process and error-related activation that suggest the possibility of compensation (32, 46). When considered in combination, these findings are consistent with the impairment of multiple cognitive control processes and the possibility that these are associated with different underlying mechanisms.

## Neuropathology and Cognitive Control Following Moderate-To-Severe TBI

Studies of TBI have investigated the relationship between activation changes during cognitive control and measures of white matter integrity obtained using DTI (18, 52). Bonnelle et al. (18) used an event-related stop-signal fMRI paradigm and DTI to examine a sample of 57 TBI patients with persistent neurological problems, of which 42 had sustained a moderate-to-severe injury, as well as a comparison group of 25 healthy subjects. Stop signal reaction time (SSRT), which provides an estimate of inhibitory processing efficiency, was slower in the TBI patients, and their accuracy was slightly lower relative to that of the comparison group. Within-group analyses using a contrast comparing correct stop trials with correct go trials revealed the expected pattern of task-related activation separately within each group, including the engagement of dorsal anterior cingulate cortex, dorsolateral PFC, right anterior insula, and several posterior cerebral areas. A between-group analysis indicated no significant activation differences within areas that are expected to activate during cognitive activity (i.e., “task positive” areas), but the TBI patients had less deactivation within default mode network (DMN) areas such as the precuneus, posterior cingulate gyrus, and ventromedial PFC. The DMN is thought to be most active during resting conditions or when there is an internal attention focus (e.g., introspective thought), but it deactivates whenever a healthy individual interacts with the environment or engages in a cognitive task (53). However, during the stop-signal task, many of the TBI patients failed to exhibit DMN deactivation and these same patients also had slower SSRT, a finding which suggests that dysfunction within DMN structures contributes to cognitive control impairment following TBI.

**Figure 3:**
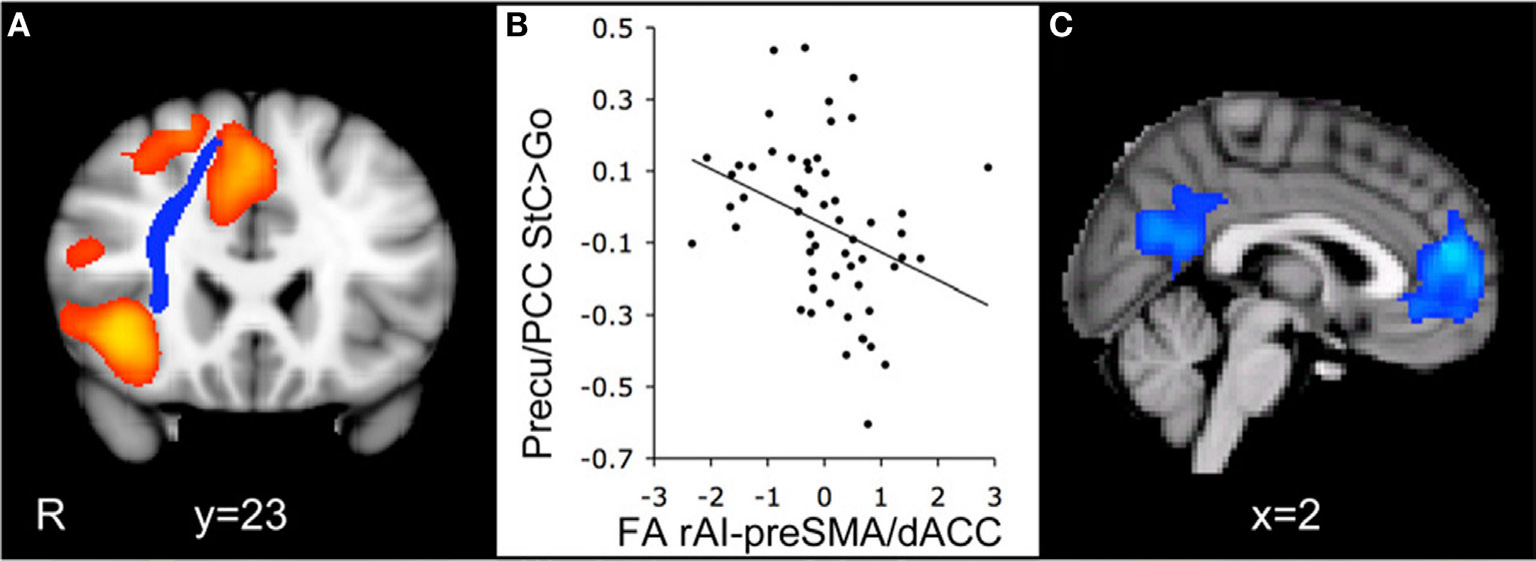
Integrity of the white matter tract connecting the right anterior insula with the pre-supplementary motor area and the dorsal anterior cingulate cortex (rAI-preSMA/dACC) predicts default mode network deactivation during the stop-signal task. **(A)** Coronal view of the rAI-preSMA/dACC tract (blue) overlaid on the activation map for the contrast comparing correct stop trials with correct go trials (StC > Go) in traumatic brain injury (TBI) patients (orange). **(B)** Fractional anistropy (FA) of the rAI-preSMA/dACC tracts in TBI patients plotted against the percent signal change within a precuneus/posterior cingulate gyrus (Precu/PCC) region of interest on correct stop trials relative to go trials. FA measures are normalized and are corrected for age and whole-brain FA. **(C)** Sagittal view of brain regions with a negative correlation between activation for the StC > Go contrast and FA within the rAI-preSMA/dACC tract. Activation is superimposed on the Montreal Neurological Institute 152 T1 template (R = right side of image) [reused with permission from Ref. (18)].

Efficient cognitive control is thought to involve coordination between the DMN and the salience network (SN), which includes structures such as the anterior cingulate cortex, pre-supplementary motor cortex, and the anterior insula (54). Activity within the SN has been said to be sensitive to the value of stimuli or events and is thought to signal the need for behavioral change, which then alters activity within other neural networks (55). According to one model of cognitive control, the right anterior insula interacts with dorsomedial frontal cortex to deactivate the DMN during cognitive activity (20, 56). Bonnelle et al. (18) examined fractional anistropy (FA), a DTI measure of white matter integrity, and in their TBI patients they found FA decreases consistent with axonal injury for several tracts connecting nodes within the SN and the DMN. They also reported that lower FA within a tract connecting structures of the SN (i.e., right anterior insula with the pre-supplementary motor area and dorsal anterior cingulate cortex) was associated with decreased DMN deactivation during stopping (see Figure 3), as well as slower SSRT. Thus, it appears that TBI-related neuropathology, in the form of decreased white matter integrity within tracts connecting nodes of the SN, may disrupt the pattern of DMN activity and contribute to impaired cognitive control.

Additional evidence for a relationship between decreased white matter integrity and cognitive control impairment following TBI was provided by Leunissen et al. (52), who used an event-related bimanual motor switching task in 22 patients with moderate-to-severe TBI and 1 with a mild injury. When compared to a group of 26 healthy subjects, these TBI patients took longer to complete motor switching and had more errors. Activation reflecting BOLD signal during switch trials relative to continue trials was greater for TBI patients within the pre-supplementary motor area and inferior frontal cortex, both of which are structures that had been specified as ROIs due to their proposed role in motor switching behavior. However, during switches, the TBI group also exhibited decreased activation within a subthalamic nucleus ROI. These investigators examined white matter integrity within six white matter tracts that connect these ROIs and found reduced FA within five. In addition, regression analyses revealed that lower FA within the tract between the left inferior frontal cortex and left pre-supplementary motor area was associated with longer switch reaction time, greater activation within the pre-supplementary motor area, and lower activation within the subthalamic nucleus. Subjects with higher activation in the pre-supplementary motor area also had lower FA within two tracts, one connecting the left pre-supplementary motor area with the subthalamic nucleus and the other connecting the right inferior frontal cortex with the right pre-supplementary motor area.

The findings of Leunissen et al. (52) are generally consistent with those of Bonnelle et al. (18), with both indicating decreased white matter integrity that is related to cortical over-activation and impaired cognitive control. The specific brain regions and tracts that exhibited these changes differed some between the studies, but the type of task and the *a priori* ROIs that were selected also differed, as well. When considered in combination, they suggest the possibility of decreased efficiency within both inter- and intra-network connections that is secondary to axonal injury. In addition to these changes in structural connectivity, functional connectivity approaches have revealed TBI-related alterations in the topological architecture of the networks during cognitive control (19), a failure to exhibit the normal pattern of functional connectivity between the right anterior insula and the DMN with either stopping or motor switching behavior (20), and a relationship between functional network alterations and both decreased FA and more severe diffuse axonal injury on structural magnetic resonance imaging (MRI) (19, 20). The convergence of results from these various modalities provides support for axonal injury as a neuropathological mechanism that disrupts interactions between nodes of the SN and DMN and contributes to impaired cognitive control. However, there is also the possibility that neuropathology underlying TBI-related deficits for one particular aspect of cognitive control, performance monitoring, may differ.

Ham et al. (46) reported that decreased performance monitoring had no relationship with white matter integrity, as measured by FA, or with either lesion location or extent. Instead, their TBI patients with low performance monitoring had increased insula activation during error-related processing and, for these same subjects, the analysis of resting state fMRI data indicated decreased functional connectivity between the dorsal anterior cingulate cortex and frontal and parietal lobe structures. There is thus the possibility that impaired performance monitoring following TBI is associated with a specific form of pathology, such as damage or dysfunction involving local cortical circuits that contribute to the BOLD signal (57), and that this may alter fMRI activation without producing changes on structural neuroimaging. As mentioned previously, the neuropathology of TBI is heterogeneous and cognitive control is a complicated construct. The impairment of different cognitive control processes may be associated with different types of TBI-related neuropathology.

## Cognitive Control-Related Activation Following Civilian (Adult) mTBI

Studies using cognitive control fMRI paradigms have also been conducted with samples consisting mostly or entirely of patients with mTBI. Smits et al. (40) employed a block design Stroop fMRI paradigm to examine 21 patients with mTBI and 12 healthy volunteers. Patients with mTBI were prospectively and consecutively enrolled through a hospital emergency department, and the imaging was performed approximately 1 month after injury. Scores from the Rivermead Postconcussion Symptoms Questionnaire (58) were used to further divide this mTBI sample into groups with moderate postconcussive symptoms (PCS) (*n* = 10) and severe PCS (*n* = 11). During the Stoop interference condition, those with severe PCS had more errors than the healthy comparison subjects and, consequently, task accuracy was included within the statistical model. An image regression analysis using an interference versus neutral block contrast and including all three groups indicated increased activation in association with higher PCS severity within the left insula, left ventrolateral PFC, and bilaterally within the precuneus and the anterior and posterior cingulate gyri. These findings were generally consistent with the results of studies of moderate-to-severe TBI, but another investigation that also used a block design version of the Stroop paradigm with mTBI patients reported no significant results (59). Terry et al. (59) studied 20 athletes with a history of multiple mTBI recruited through a university setting and compared these with a sample of 20 healthy volunteers. Their assessment was performed an average of 19.6 months after the most recent injury, and there were no between-group differences for task accuracy, reaction time, or for whole brain and ROI image analyses. These investigators attributed the lack of significant activation imaging findings during the Stroop paradigm to recovery of function during the relatively long post-injury interval.

Different versions of the Stroop paradigm have been used to examine the neural substrate of cognitive control and one particularly sophisticated approach was employed by Mayer et al. (60, 61), who adapted a numeric version of the task to study multisensory (i.e., visual and auditory) selective attention while manipulating the level of cognitive and perceptual load. During this event-related paradigm, congruent and incongruent numbers were simultaneously presented through both visual and auditory modalities at either a low or a high presentation rate. Each block of stimuli was preceded by an auditory cue, consisting of a single word which instructed the subject not to respond (“NONE”), or to press a button in response to only the visual (“LOOK”) or auditory (“HEAR”) stimuli. In their first study, they examined 22 patients who had sustained an mTBI sometime within the previous month and compared these to 22 healthy volunteers. Performance accuracy for both groups approached ceiling and there were only minimal differences for reaction time. There were no between-group activation differences for an incongruent versus congruent contrast within areas thought to mediate cognitive control, but during rapid stimulus presentation the mTBI group failed to exhibit task-induced deactivation within the DMN. In addition, the experimental design allowed these investigators to examine how top-down control, directed in response to instructional set (i.e., which sensory modality to attend to), enhances or suppresses neuronal responses within sensory and heteromodal areas. While attending to the visual modality their healthy comparison group exhibited signal increases within bilateral dorsolateral PFC and the visual stream. However, these adaptive signal changes, referred to as attention-related modulations, were not found while mTBI patients were attending to the visual stimuli. In a follow-up study, these investigators again reported finding no evidence of dysfunction within the cognitive control network during the numeric Stroop paradigm, but abnormal neural responses were found within inferior parietal and unisensory cortex, and these had only partially resolved when the same mTBI patients were rescanned 4 months later (61). When considered in combination, these results suggest the possibility that neural function associated with cognitive control is not consistently altered at the mild level of severity, or that it recovers relatively quickly, while subtle changes involving sensory functions may be more persistent (61).

## Cognitive Control-Related Activation Following Military TBI

Explosive blast is a common cause of military TBI and post-deployment surveys and clinician-confirmed diagnoses have indicated that about 90% of these injuries are mild (62, 63). The actual mechanism of injury is often unclear because explosions can produce effects through rapid changes in atmospheric pressure (i.e., blast over- and/or under-pressurization), as well as mechanical impact when the individual is struck by debris or thrown against structures or the ground (64). Furthermore, the association of military mTBI with post-deployment PCS is non-specific because posttraumatic stress disorder (PTSD) symptoms associated with combat exposure are also predictive of PCS in the chronic phase of outcome (62, 63). The frequent presence of comorbid conditions such as PTSD and depression, complicated mechanisms of injury, the possibility of repeated blast exposure with multiple mTBIs, and the lack of accurate medical records or other documentation for injuries that were sustained during combat deployment pose significant challenges for both clinicians and researchers (65). However, it is critical that research address relationships among these various factors, and a small number of fMRI studies have investigated cognitive control following deployment-related mTBI.

Using an event-related version of the Arrows Task fMRI paradigm, Scheibel et al. (66) examined 30 veterans and active duty service members who had been deployed to Afghanistan or Iraq. Fifteen of these had sustained at least one blast-induced mTBI with an average post-injury interval of 2.6 years and, in addition, six reported a history of multiple blast-related mTBIs. The other 15 subjects had also completed deployment but had not sustained a TBI or been exposed to blast. There were no between-group differences for task accuracy, but non-significant trends favored slightly slower responding for the mTBI group during both congruent and incongruent trials. For the contrast of incongruent versus congruent trials, the mTBI group had greater activation relative to the comparison group within the anterior and posterior cingulate gyri, MFC, and posterior cerebral regions thought to be involved in visual and visual–spatial functions. However, this over-activation was also noted to be more extensive after statistically controlling for response time and symptoms of PTSD and depression. Higher scores on the PTSD Symptom Checklist-Civilian version (PCL-C) (67) were related to decreased activation within both groups (66) and, when examined in the comparison subjects alone, PTSD symptoms were noted to be associated with extensive deactivation throughout large areas of the brain (68). This pattern of suppressed activation in the present of PTSD symptoms differs from the over-activation that was found with blast-related mTBI, as well as that reported previously with moderate-to-severe TBI in studies of civilians that had used the block design version of this same task (27, 35, 36). These findings are consistent with different effects of TBI and PTSD on cognitive control-related brain activation, probably reflecting different underlying neural mechanisms associated with physical and psychological trauma, and they illustrate the importance of addressing comorbid disorders and their symptoms in fMRI research examining post-deployment populations.

Another study examined cognitive control following blast-related mTBI and found a relationship between brain activation and both somatic symptoms and comorbid major depressive disorder (MDD) (69). Matthews et al. (69) used an event-related stop signal fMRI paradigm with a group of 15 subjects who had experienced loss of consciousness (LOS) following blast-related TBI and compared them to 12 who had reported only alteration of consciousness (AOC). Their stop signal task was individualized to ensure comparable difficulty across participants and the analysis used the contrast of hard versus easy inhibition trials to compare mTBI subjects with LOC and AOC. An additional diagnosis of MDD was noted to be more common in the patients with LOC, so the presence of comorbid MDD was included within the statistical model. A whole brain image analysis revealed a significant interaction, with the LOC group exhibiting less activation relative to the AOC group within a cluster in the left ventromedial PFC, but only during the easy inhibition trials. Within the LOC group, there was also a significant positive correlation between activation in the ventromedial PFC and self-ratings of somatic symptoms, as assessed by the Patient Health Questionnaire-15 (70). These investigators speculated that hypoactivity within the ventromedial PFC may lead to impaired self-awareness and the under-reporting of somatic complaints. In addition, patients with MDD were found to have reduced inhibition-related activation within three relatively large clusters that included the anterior cingulate cortex, superior frontal gyrus, and the cerebellum. This latter finding is similar to what has sometimes been reported in subjects with PTSD symptoms (68), suggesting that both PTSD and MDD may alter the activation pattern following military TBI.

Fischer et al. (71) also used an event-related stop signal task to study brain activation following military, blast-related TBI, but they went further and included a comparison with civilian TBI. Their design included four groups: a military TBI group with 21 subjects (18 mild and 3 moderate TBI), a military control group with 22 uninjured subjects, a civilian TBI group with 21 subjects (19 mild, 2 moderate TBI), and a civilian control group which included 23 orthopedic injury patients. All of the TBI subjects had sustained their injuries 1–6 years earlier, and the civilian groups had better task performance than the military groups. During correct inhibitions (i.e., correct inhibition trials minus correct go trials), the TBI patients had decreased activation, relative to their control groups, within the DMN and in regions thought to be associated with inhibitory control, such as the anterior cingulate gyrus. However, a more complex relationship was noted for activation during incorrect inhibitions (i.e., incorrect inhibition trials minus correct go trials) within bilateral inferior and left superior temporal cortex, the caudate, and the cerebellum. For these areas, the military TBI patients had increased activation relative to their comparison group, while the civilian TBI group exhibited decreased activation when compared to the civilian orthopedic injuries. These investigators stated that blast-related TBI may have elevated activation associated with inhibitory failure within these particular brain areas, which they said are involved in the cognitive/emotional interpretation of negative feedback, while in cases of mechanical brain injury (i.e., civilian TBI) the failure to inhibit appears to be associated with reduced activation within these same structures. Thus, they concluded that blast-related TBI may have a unique effect on brain function during inhibitory failures that is distinguishable from mechanical TBI.

## Cognitive Control-Related Activation Following Pediatric TBI

A couple of functional neuroimaging studies have addressed cognitive control following pediatric TBI. The Counting Stroop paradigm was used by Tlustos et al. (72) to study 11 adolescents, with a mean age of 15.7 years, who had sustained a TBI requiring an overnight hospital admission. All of the injuries had occurred at least 1 year earlier and eight of these were classified as complicated mild, with a GCS total score of 13–15 accompanied by abnormalities on imaging, while one subject had a severe TBI and the other two injuries were of moderate severity. A comparison group of 11 typically developing adolescents was matched to the injured group on gender, handedness, and race/ethnicity and had a mean age of 15.2 years. The two groups had equivalent accuracy during scanning, and there were no between-group differences for reaction time during the interference condition, but the TBI group was slower during the neutral (i.e., non-interference) condition. A whole brain image analysis contrasting these two conditions revealed greater activation for the TBI group within the dorsal anterior cingulate gyrus and right frontal and parietal areas, including dorsolateral PFC. These findings were said to be generally consistent with those of a number of previous studies that had used similar fMRI paradigms and found increased activation in adults with TBI [e.g., Ref. (35, 36, 40)].

Another study (28) examined 13 slightly younger mTBI patients (mean age = 13.3 years) using the Tasks of Executive Control (TEC) (73), which is an fMRI paradigm that includes an *n*-back working memory procedure and an embedded go/no-go task. During their TEC paradigm, the subject views the sequential presentation of stimuli, which consist of colored images of common objects (e.g., tree, dog), and uses a button press to place the object into one of two toy boxes based on the rules for the current condition. For example, for the 1-back condition, the target is any object that is seen twice in a row, and these are to be placed in a toy box on the left side of the display, while non-targets are placed in a toy box on the right. The paradigm includes 0-, 1-, and 2-back conditions. However, in addition to these standard *n*-back conditions, there is an equivalent set of conditions that also include inhibit cues, which consist of a box that is displayed around a small proportion of the objects to indicate that no response should be made. The TEC was used to acquire data from the mTBI subjects and a comparison group of 13 typically developing children who were matched on age, gender, and IQ. The mTBI group had been injured between 8 and 82 days earlier (mean = 29 days), and all TBI patients were still symptomatic at the time of examination, as determined by a parent report score of 11 or greater on the Post-Concussion Symptom Inventory (74). Unfortunately, due to a problem with the fMRI paradigm, TEC performance data were not available. In a whole brain image analysis, there were no significant between-group differences for the contrast of the 2-back interference condition versus the standard 2-back condition, but a similar comparison at the 1-back level indicated greater inhibitory control activation for the mTBI group bilaterally within the posterior cerebellum. An ROI analysis also found that injured subjects had increased activation within the cerebellum and, for the mTBI group, there was a significant negative correlation between cerebellar activation and scores from the metacognitive index of the BRIEF.

Findings from the TEC fMRI paradigm differ from those of Tlustos and colleagues (72), who reported a pattern of over-activation with the Counting Stroop paradigm that was more similar to that which has been found in a number of studies of cognitive control with adult TBI. However, examination of the standard working memory contrasts from the TEC did not indicate any between-group differences, which also differs from the results of neuroimaging studies of working memory following TBI in both adult (75, 76) and pediatric populations (77, 78). Krivitzky et al. (28) attributed these differences to developmental factors, stating that nodes within working memory networks may be recruited differently in children and adults, particularly when there is an additional demand for inhibitory control. Although this is a possibility, each of their inhibition conditions included a relatively small number of trials, one of these levels was reported to be too difficult for some of the children, and they were unable to examine the relation between task performance and activation. Consequently, it is also possible that issues with task performance or low statistical power may have limited their ability to detect between-group differences and, in the absence of such complications, they may have found more extensive over-activation during cognitive control in the children with mTBI.

## Conclusion and Future Directions

Although the results of studies of moderate-to-severe TBI vary, when considered in combination there is evidence for over-activation that includes and extends beyond brain areas that are thought to be directly involved in cognitive control. This general finding is consistent with other research that has documented increased activation in clinical samples using paradigms engaging other cognitive functions (24, 25), including working memory, especially when task performance approaches that of normal comparison subjects (22). Increased cognitive control-related activation following TBI is most clearly found in association with severe injury, as shown by studies examining predominantly severe TBI samples (27, 30, 35, 36), in comparisons among different severity subgroups (36), and with image regression analyses that have included acute injury measures such as the GCS (32, 35, 36). Activation in TBI patients also appears to be greater as TOT increases (29, 32), perhaps reflecting an inability to shift from effortful top-down cognitive processes to those that are more automatic, greater vulnerability to cognitive fatigue, and underlying neural inefficiencies that require the TBI patient to exert more effort to maintain adequate performance over time.

Another finding with cognitive control paradigms following TBI is a failure to deactivate components of the DMN (18), including the posterior cingulate gyrus and precuneus, and these same brain areas have also been reported to exhibit over-activation relative to comparison subjects (36). Thus, dysfunction within the DMN is probably one of the most common findings among functional neuroimaging studies investigating cognitive control after TBI. The DMN is one of several distributed neural networks that interact to facilitate efficient information processing, and in healthy individuals it has been shown to be most active while at rest, in association with introspective thought, or during the evaluation of certain types of social information (e.g., self-other referencing) (53, 79). Another network is the central executive network (CEN), for which the dorsolateral PFC is a key node, and this network is thought to be involved in top-down processes associated with task-set maintenance and goal achievement (11, 12, 80). During the performance of cognitively demanding tasks, the CEN typically exhibits activation increases, while at the same time activation within the DMN has been noted to decrease (55, 80). The SN also contributes to cognitive control and its nodes include the anterior cingulate cortex, pre-supplementary motor cortex, and the anterior insula (54). This network has been said to monitor the value of stimuli or events and to deactivate the DMN, allowing attention to shift away from an internal focus, so that it can address external cognitive demands, as well as to signal the CEN that control needs to be implemented to increase effort or adjust the response strategy (15, 55). Each individual brain structure may have a critical role, but it is the integrity of connections within and between the networks that allow these nodes to interact effectively. Some connections allow rapid communication between distant structures, such as the anterior insula and anterior cingulate cortex (17, 55), and these longer fiber tracts may be especially vulnerable to disconnection due to TBI.

The neuropathology associated with TBI is heterogenous (2), but multifocal or diffuse axonal injury is a common feature and this may decrease the efficiency of both inter- and intra-network connections (18, 44). The results of studies utilizing DTI and event-related fMR provide support for posttraumatic axonal injury as a neuropathological mechanism that reduces white matter integrity within fiber tracts, which alters activation patterns and functional connectivity during cognitive control, and impairs task performance (18–20, 52). It seems likely that the partial disconnection of structures within distributed neural networks reduces their efficiency and increases the demand on neural resources. However, impaired performance monitoring following moderate-to-severe TBI is associated with changes in functional connectivity and increased error-related activation within the insula, but not structural changes in MRI (46). There is the possibility that different types of pathology such as axonal injury, focal lesions, and the disruption of local cortical interneurons may each make their own contribution to activation changes and cognitive impairment following TBI (81). Research is needed to address the contribution of various types of TBI-related neuropathology to altered cognitive control following moderate-to-severe TBI, including the impairment of specific cognitive processes.

Studies utilizing cognitive control fMRI paradigms have also assessed the effects of mTBI, and these have reported a combination of complicated findings that have included increased activation (40, 66, 71, 72), decreased activation (69, 71), and no significant between-group differences (59). In general, there is preliminary evidence for over-activation with some similarity to what has been found with more severe injuries, but such findings may be limited to mTBI patients who are currently symptomatic, seeking treatment, or also have a focal lesion on structural brain imaging (40, 66, 72). In one study, there was a failure to deactivate the DMN at 1 month post-injury, but not 4 months later (60, 61). Large differences in the injury to assessment interval may contribute to inconsistent findings among various studies (61), especially since the symptoms of post-concussive syndrome often resolve over a period of weeks or months (82), and there is currently insufficient data to determine whether activation changes persist after the resolution of clinical symptoms. In addition, the total number of studies that have been completed is small, and these have examined several different mTBI populations, some of which are likely to introduce additional variability due to developmental factors, the possibility of multiple injuries, or comorbid conditions that influence brain activation in their own way. Thus, there is clearly a need for studies with well-defined samples and adequate controls for comorbid disorders, different injury characteristics, time since injury, and severity of post-concussive symptoms (38).

A number of mechanisms have been proposed to account for over-activation and other alterations in cognitive control-related activation following TBI, including compensation, increased engagement of normal processes to maintain adequate performance, and decreased neural efficiency requiring greater effort and resource utilization (25, 38, 83). There is some support for each of these and they may not be mutually exclusive, especially since TBI is heterogeneous and different types of neuropathology or injury to specific fiber tracts may impair different processes associated with cognitive control. One critical issue for the interpretation of fMRI findings and for relating activation changes to underlying mechanisms is performance (22). If activation changes following TBI are not related to improved performance then any argument in favor of a compensatory mechanism would appear to be weakened (25). However, many experimental designs manipulate performance to facilitate the comparison of activation patterns and, in addition to reducing between-group differences, such procedures are also likely to decrease the range and variability of the performance measures. These alterations to the distribution of accuracy and reaction time variables may reduce the strength of any coefficients that include them (84), including those calculated using image regression procedures. An alternative approach may be to perform such regression analyses using variables that are not obtained as part of the fMRI data acquisition procedures, such as clinical symptom measures, disability ratings, and cognitive performance scores acquired through procedures other than the fMRI paradigm. A few studies have used such an approach and have provided some initial data favoring a relationship between increased activation and scores on executive function measures, a finding that has been interpreted as supporting the possibility of a compensatory mechanism (28, 32). In addition, studies examining the relationship between activation and cognitive or symptom measures may contribute information regarding the external validity and relevance of the functional neuroimaging technique, itself.

Research using fMRI to study cognitive control following TBI may also benefit from a number of other improvements, including some to address basic statistical limitations. For example, many studies had sample sizes that were below commonly accepted recommendations or included an insufficient number of trials to support the various contrasts that were performed, especially when correct and incorrect responses were separated (85–87). Another statistical issue involves error correction and the statistical thresholds that have been used for whole brain analyses. According to Eklund et al. (88), the procedures associated with some image analysis software applications were too liberal and family-wise error rates were above the expected 5% for cluster-wise inference. Recommendations have since been published for addressing these error correction issues (89). Additional possibilities for future research may include the examination of new topics that are relevant for cognitive control or that may help explain the high degree of between-subject variability that is often found with functional neuroimaging data. For example, fMRI studies investigating cognitive control following TBI have not yet examined the role of genetic factors or reward mechanisms. Recent approaches to cognitive control place some emphasis on reward circuitry (13) and an examination of effort, motivation, and incentives may provide useful information that could be relevant for cognitive rehabilitation and other interventions for TBI.

## Summary

Relatively few functional neuroimaging studies have addressed cognitive control following TBI, and these have often suffered from significant methodological limitations, but there is currently some evidence for alterations in the neural substrate that supports this complex executive function. Activation associated with the engagement of cognitive control appears to be elevated and more dispersed following TBI, especially at the severe level of injury, and there may also be a failure to deactivate structures within the DMN. Examination of structural and functional connectivity data suggests that at least some of these changes may be related to decreased white matter integrity following TBI. The results of studies that have focused on mTBI have been more variable and are difficult to interpret, but there may be mTBI populations with activation changes that overlap with those found following more severe injuries, including symptomatic mTBI patients and those with acute injuries. Future research may benefit from the use of well-characterized TBI samples, examination of neuropathology data from other imaging modalities, inclusion of external performance variables and clinical symptom or disability measures, and the use of experimental designs that allow the examination of multiple processes associated with cognitive control.

## Author Contributions

The author confirms being the sole contributor of this work and approved it for publication.

## Conflict of Interest Statement

The author declares that the research was conducted in the absence of any commercial or financial relationships that could be construed as a potential conflict of interest.
